# Role of High Energy Breakfast “Big Breakfast Diet” in Clock Gene Regulation of Postprandial Hyperglycemia and Weight Loss in Type 2 Diabetes

**DOI:** 10.3390/nu13051558

**Published:** 2021-05-05

**Authors:** Daniela Jakubowicz, Julio Wainstein, Shani Tsameret, Zohar Landau

**Affiliations:** 1 Diabetes Unit, Wolfson Medical Center, Sackler Faculty of Medicine, Tel Aviv University, Holon 58100, Israel; vainstein@wolfson.health.gov.il; 2 Institute of Biochemistry, Food Science and Nutrition, The Robert H. Smith Faculty of Agriculture, Food and Environment, The Hebrew University of Jerusalem, Rehovot 76000, Israel; shanitsameret@gmail.com; 3 Pediatric Division, Barzilai Medical Center, Faculty of Health Sciences, Ben-Gurion University of the Negev, Beer-Sheva 84000, Israel; landau.zohar@gmail.com

**Keywords:** clock genes 2, big breakfast 3, PPHG 4, T2D 5, circadian rhythms

## Abstract

Postprandial hyperglycemia (PPHG) is strongly linked with the future development of cardiovascular complications in type 2 diabetes (T2D). Hence, reducing postprandial glycemic excursions is essential in T2D treatment to slow progressive deficiency of β-cell function and prevent cardiovascular complications. Most of the metabolic processes involved in PPHG, i.e., β-cell secretory function, GLP-1 secretion, insulin sensitivity, muscular glucose uptake, and hepatic glucose production, are controlled by the circadian clock and display daily oscillation. Consequently, postprandial glycemia displays diurnal variation with a higher glycemic response after meals with the same carbohydrate content, consumed at dusk compared to the morning. T2D and meal timing schedule not synchronized with the circadian clock (i.e., skipping breakfast) are associated with disrupted clock gene expression and is linked to PPHG. In contrast, greater intake in the morning (i.e., high energy breakfast) than in the evening has a resetting effect on clock gene oscillations and beneficial effects on weight loss, appetite, and reduction of PPHG, independently of total energy intake. Therefore, resetting clock gene expression through a diet intervention consisting of meal timing aligned to the circadian clock, i.e., shifting most calories and carbohydrates to the early hours of the day, is a promising therapeutic approach to improve PPHG in T2D. This review will focus on recent studies, showing how a high-energy breakfast diet (Bdiet) has resetting and synchronizing actions on circadian clock genes expression, improving glucose metabolism, postprandial glycemic excursions along with weight loss in T2D.

## 1. Introduction

Postprandial hyperglycemia (PPHG) in type 2 diabetes (T2D) strongly contributes to glycated hemoglobin (HbA1c) values [1]. It is linked to increased risk for the development of cardiovascular complications, even when glycemic control is restored [2,3]. Further, PPHG leads to a progressive decline of β-cell function and deficient and delayed early postprandial insulin response [4,5,6]. Hence, the reduction of glycemic peaks is an essential “target” in the treatment of T2D to mitigate the decline of β-cell secretion and prevent cardiovascular complications [2,6,7].

The circadian clock temporally coordinates the metabolism over a 24-h period to anticipate daily recurring feeding-fasting cycles and optimize the metabolic efficiency at an appropriate time of the day, thereby preventing metabolic dysregulation [8,9,10,11,12,13,14,15,16]. Most of the hormonal and enzymatic functions controlling PPHG, i.e., secretion of insulin [17,18,19], glucagon-like peptide-1 (GLP-1) [20], GLUT-4 expression in skeletal muscle [21,22], and hepatic glucose production [23,24]; are regulated by the circadian clock and display diurnal variations.

The circadian clock is controlled by light/dark signals and other external inputs such as meal timing or food availability [12,13,14]. The insulin sensitivity, β-cell responsiveness, GLUT-4 activity, and muscular glucose uptake are all enhanced in the early hours of the day compared to dusk or evening [8,16,17,18,19,20,21,22,23,25,26,27,28,29,30,31,32,33]. Therefore, the metabolism is optimized for food intake in breakfast, while the evening and nighttime are optimal for fasting and sleep [9,34,35,36,37]. Indeed, postprandial glycemia displays a clear circadian pattern, with higher glycemic response after meals with the same carbohydrate content, consumed at dusk compared to the morning, both in healthy [23,27,29,33,38,39], and T2D individuals [14,15,25,35,40].

Meal timing exerts a critical influence on peripheral clocks involved in postprandial glycemia [14,15,41,42,43,44].

Circadian misalignment, with day/night cycle, often imposed in modern society, like shift workers, skipping breakfast, snacking all day, including in the evening hours, are associated with disrupted clock gene expression and linked with aberrant metabolic responses, weight gain, PPHG, increased risk for T2D [12,27,34,37,42,45,46,47,48,49,50,51,52], and cardiovascular complications [53]. Breakfast skipping is also linked to a significant increase in HbA1c even without overeating in the evening [47].

Asynchrony of the circadian clock is central in the pathophysiology of T2D [12,13,54]. Lower transcripts of clock gene expression in T2D are linked to insulin resistance, delayed β-cell secretion and reduced β-cell proliferation [12,27,34,37,48,55], PPHG, and increased HbA1c [15,36,43,54]. Moreover, breakfast’s omission in T2D patients causes further disruption in clock gene expression, and it is linked to PPHG and delayed and deficient early insulin and GLP-1 responses after subsequent meals [15,43,49]. In contrast, meal-timing pattern, aligned with the circadian clock, consuming high in energy breakfast, exerts a powerful effect on the clock network temporal synchronization, thereby improving the postprandial glycemic responses across the day in healthy and T2D patients [15,35,39,43,56].

Therefore, breakfast consumption might be critical in T2D for the achievement of metabolic homeostasis and improvement of PPHG [14,15,23,24,25,26,27,28,29,30,31,32,33,34,35,36,37,38,39,40,43,44,45,46,47,48,49,50,51,52,53,54,55,56]. Hence, resetting clock gene expression through a diet intervention consisting of meal timing aligned to the circadian clock is a promising therapeutic intervention approach to improve PPHG [12,15,27,43].

In this review we discuss recent studies showing how the high energy breakfast diet designed as “big breakfast diet” (Bdiet) has resetting and synchronizing actions on clock gene expression, improving glucose metabolism, postprandial glycemic excursions, and bodyweight in T2D.

## 2. Circadian Clock Regulation of Metabolism

### 2.1. Central and Peripheral Clocks

The diurnal variation of the hormonal and enzymatic functions related to glucose metabolism and postprandial glycemia is synchronized by the circadian clock [13,14,16].

The central or “Master Clock” is found in the hypothalamus in the suprachiasmatic nucleus (SCN). The clock genes located in SCN are synchronized by light signals and generate the body endogenous ~24-h rhythm. Other clock genes are disseminated in almost all the peripheral tissues, i.e., muscle, liver, intestinal L-cells, and in β-cells [12,13,14,57,58,59]. The clock genes located in peripheral tissues are coordinated by signals coming from the SCN [59,60]. However, they are mostly entrained to the time of food intake and food availability [18,21]. It allows the peripheral clocks to anticipate the secretion of metabolic hormones and enzymes just before the food consumption at a specific hour of the day [12,13,14,15,20], leading to synchronized, hormonal, digestive, absorptive, and metabolic functions [8,9,13,14,16,17,18,19,20,21,22,23,55]. The synchronization of peripheral clock genes to the time of food intake is achieved in a tissue-specific fashion through multiple postprandial signals, i.e., absorbed nutrients, post meal glucose excursions, β-cell, and GLP-1 secretion [13,52,58,60,61,62,63,64,65,66,67].

**Figure 1:**
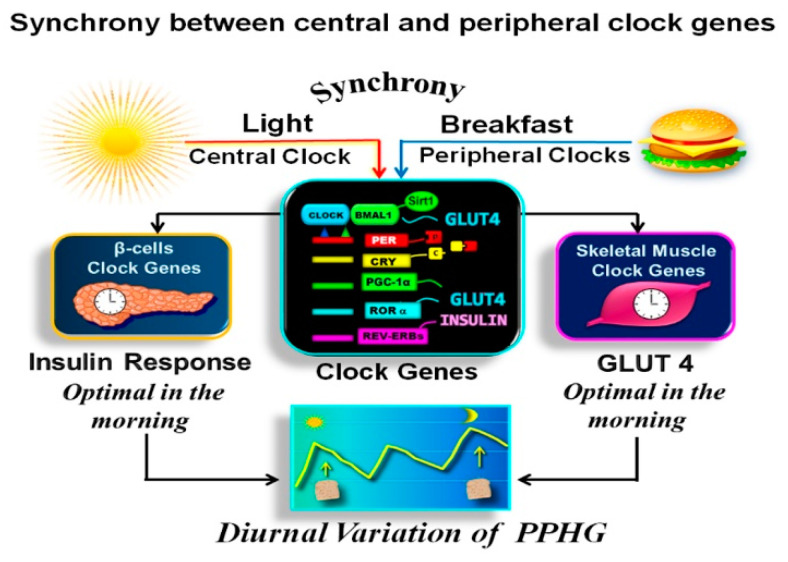
Synchronization between central and peripheral clock genes. In the above illustration, we observe that the breakfast in synchrony with downlight “turns on” the clock, activating the *CLOCK:BMAL1* complex. It drives the transcription of *PERs*, *CRYs*, *REV-ERBs*, *RORs* genes, *PPARγ* coactivator 1α (*PGC-1α*), *SIRT1*, and other transcriptional elements promoting downstream expression of several proteins encoded by tissue specific “clock-controlled genes,” relaying the clock information to cellular processes. As a result, β-cells insulin response and muscular GLUT-4 activity are optimal in the early hours of day. Hence the glucose response after isocaloric meals is significantly higher in the evening than in the morning.

Noteworthy is that the first meal of the day, i.e., breakfast, exerts a more powerful resetting effect on the clock network than other meals, underscoring the damage caused by the absence or delayed breakfast on the clock regulation of glucose metabolism and PPHG [43,49,51,57,68] (Figure 1).

### 2.2. Molecular Mechanism of the Circadian Clock-Driven Metabolism

The molecular clock mechanism is identical in central and peripheral clocks. It consists of self-sustained transcriptional-translational feedback loops [12,55,58,59]. The transcriptional activators *CLOCK* (circadian locomotor output cycles protein kaput) and *BMAL1* (brain and muscle ARNT-like 1) act as positive elements in the feedback loop. *CLOCK:BMAL1* heterodimer drive the transcription of periods (*PERs*) and cryptochromes (*CRYs*) genes. The resulting PER and CRY proteins dimerize in the cytoplasm. After ~24 h, they are translocated back into the nucleus to interact with the *CLOCK:BMAL1* complex, directly suppressing their own transcription, thus generating a cycle that recurs every ~24 h [12,55,58]. In a secondary regulatory loop, *CLOCK:BMAL1* mediates the transcription of the repressor nuclear receptor *REV-ERBα* and one promoter gene, the retinoic acid receptor-related orphan receptor (*RORα*), maintaining further the circadian (24 h) oscillation of the clock [14,58].

The *CLOCK:BMAL1* heterodimer also mediates the transcription of tissue-specific output genes, *PERs*, *CRYs*, *REV-ERBs*, and *RORs* clock genes, along with PPARγ coactivator 1α (*PGC-1α*), and *SIRT1* and other transcriptional elements, which promote downstream expression of several tissue-specific proteins, hormones and enzymes, relaying the clock information to cellular processes, like β-cell secretion, GLUT-4 activity, hepatic glycogenolysis, and gluconeogenesis [12,24,52,55,58].

### 2.3. Circadian Clock Regulation of Glucose Metabolism and Postprandial Glycemia

*BMAL1:CLOCK* complex plays a critical role in the transcription of tissue-specific elements, which regulate the circadian processes involved in glucose homeostasis and postprandial glycemia [43,59]. *BMAL-1*, *RORα*, and *SIRT1* positively regulate the circadian β-cells secretion [18,20,23,60], insulin sensitivity [62,68], muscular GLUT-4 activity, and glucose uptake [21], and β-cell replicative capacity and survival [13,60,62,69]. *BMAL-1* and *RORα* integrity are also necessary for circadian GLP-1 secretion of in the intestinal L-cells [63]. In the liver, the expression of *REVERBα* and *RORα, SIRT1* and *PGC-1α*, modulates the gluconeogenic enzymes, and the rhythms of hepatic glycolysis pathway [52,58,64,65,66,67,68].

*BMAL1*, *CRY2*, *CRY1*, and *PER2*, through posttranslational regulation of cAMP signaling, reduces the glucagon-stimulated hepatic glucose production [66], and coordinate the nocturnal oscillation of hepatic glucose output; glycogenolysis in the first part of night and gluconeogenesis in the second part, before waking up [24,66].

Adenosine monophosphate-activated protein kinase (*AMPK*), plays a crucial role in the clock regulation of glucose metabolism [16]. Upregulation of *AMPK* expression significantly enhances GLUT-4 translocation and muscular glucose uptake, ensuring metabolic efficiency and improving postprandial glucose and insulin responses [66,67,68,69]. *AMPK* exerts a positive effect on *SIRT1*, associated with beneficial effects on insulin sensitivity and β-cells viability [52,58,69,70,71,72].

### 2.4. Disrupted Clock Genes Expression in Type 2 Diabetes

Asynchrony of clock gene expression is essential in the pathophysiology of obesity, metabolic syndrome, and T2D [34,71,72]. It is also associated with circadian misalignment of meal timing or sleeping hours like in shift-workers [34,71,72]. Disrupted clock gene expression is associated with reduced and delayed β-cell response, insulin resistance, and a low rate of β-cell replication [12,27,37,55]. Deficient *BMAL1* and *CRY2* expression in T2D is associated with PPHG and higher HbA1c levels [15,36,43,54].

### 2.5. Synchronization between Central and Peripheral Clocks

For the functionality of the circadian clock, the individual clocks must be synchronized one to another and with the external environment [38,52]. This coordination is achieved when the feeding/fasting cycle is aligned with the day/night cycle [9,10,11,38]. Therefore, both stimuli, “light” and “food”, should occur simultaneously “in synchrony” (Figure 1). As breakfast consumption has a powerful resetting effect on the clock network, the temporal synchronization between breakfast and downlight is critical for achieving metabolic homeostasis [43,49,51,57,58].

In Figure 1 is shown how the breakfast in synchrony with downlight “turns on” the clock gene machinery in the early morning. This further regulates the clock-controlled output genes relaying the clock information downstream to the tissue-specific proteins and the rhythms of cellular processes [8,55,58].

### 2.6. Asynchrony between Central and Peripheral Clocks

**Figure 2:**
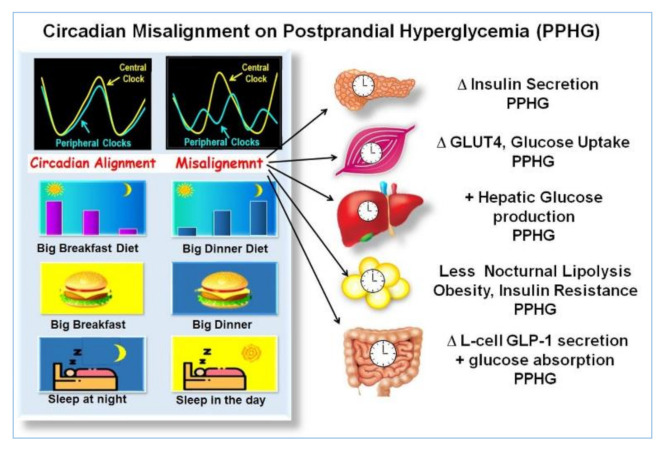
Effects of misalignment on postprandial hyperglycemia (PPHG). The illustration, shows, how eating and sleeping at hours not aligned with the circadian clock, i.e., small breakfast, big dinner, sleeping during the day, etc., produce a misalignment between central and peripheral clocks and disrupted clock gene expression. It is associated to deficient β-cell secretion, GLUT-4 activity, muscular glucose uptake, increased hepatic glucose output, adipogenesis, reduced lipolysis, insulin resistance, alteration of GLP-1 secretion, and increased intestinal glucose absorption. All of which may result in worsening of PPHG “Adapted with permission” [9].

Eating and sleeping out of synchrony, delaying the first meal or increasing the frequency of the meals, with calories and CH uniformly spread across the day, including evening hours assigned to rest, promote the uncoupling or desynchronization between the peripheral and the central clock genes, and disrupted regulation of metabolic processes. This misalignment may result in altered thermogenesis, weight gain, increased lipids, insulin resistance, fatty liver, and worsening of postprandial glycemia, as it was shown in preclinical studies [41,42,44,48,49,50,51], clinical studies in non-diabetic [9,10,11,30,39,43] and in T2D individuals [15,43,45,46,47] (Figure 2).

## 3. Effect of High Energy Breakfast “Big Breakfast Diet” on Resetting Clock Gene Expression and Reduction of PPHG in T2D

The circadian clock regulation of PPHG is influenced by the meal timing schedule [14,41,42,44]. Breakfast skipping and over-eating in the evening led asynchrony of the circadian clock, and is linked to weight gain, PPHG, and diabetes [43,48,49,50].

Several recent reports suggest that eating in synchrony with the circadian clock by shifting more energy and CH to the morning hours (i.e., high energy and CH breakfast), and reducing energy and CH consumption in the evening hours, facilitate weight loss, improve postprandial glycemia, and reduce appetite and craving in metabolic syndrome and in T2D, compared to the inverse pattern, i.e., “high in energy and CH dinner” and reduced breakfast [15,27,30,35,39,40,61,73,74,75,76,77]. Clinical and epidemiological studies have shown that late meals are linked to obesity and T2D [39,40,47]. A diet intervention not aligned with the circadian clock by shifting calories and CH to later hours of the day is associated with less weight loss and higher postprandial and overall glycemia among obese [39,40] and in T2D individuals [15,35].

### 3.1. Effect Skipping Versus Eating Breakfast, on Clock Gene Expression and PPHG

**Figure 3:**
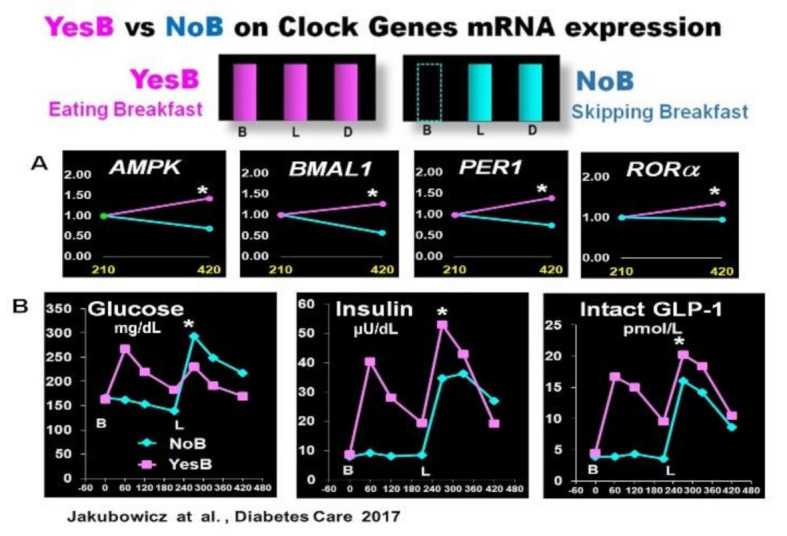
Effect of YesB vs. NoB on clock gene expression and postprandial glucose, insulin, intact GLP-1 response after breakfast and lunch in T2D. (**A**): Clock gene expression: Blood samples were collected 3.5 h after breakfast (YesB) or no breakfast (NoB) (time point 210 min) and 3.5 h after lunch (time point 420 min) Asterisks denote statistical differences (*p* < 0.05) between time point 210 min and time point 420 min. Data are means ± SE. (**B**): Line charts of glucose, insulin, and intact GLP-1 postprandial responses on YesB and NoB days: Breakfast (B) was given to the YesB group at time point 0. Lunch (L) was given to both groups at time point 210 min. Asterisks denotes statistical differences between YesB and NoB at a specific time point. Data are means ± SE. “Reproduced and adapted with permission” [43].

In two crossover studies in T2D patients, we explored whether skipping breakfast in a single day (NoB) versus another day consuming high energy and CH breakfast (YesB) influences the clock gene expression and the PPHG after subsequent isocaloric meals. [43,56]. Breakfast skipping (NoB) acutely disrupts clock gene expression after lunch [43]. The absence of breakfast (NoB) down-regulated the mRNA expression of *AMPK* and *BMAL1*, *PER1* and *RORα* expression, and this clock gene disruption in NoB day was associated with significantly higher postprandial glycemic response and deficient and delayed insulin, and intact GLP-1 postprandial secretion after lunch [43] (Figure 3). In contrast, high energy and CH breakfast consumption in YesB day led to an overall increased expression of these key metabolic clock genes, i.e., *BMAL1*, *PER1*, *RORα*, and *AMPK* [43]. This resetting effect on clock genes mRNA expression in YesB day, was associated with significant reduction of postprandial glycemic response and enhanced and faster insulin and GLP-1 response after subsequent lunch. It suggests that the upregulation of these pivotal clock genes in the YesB day led to the improvement of PPHG [35,43,56] (Figure 3).

This research showed that just a single day of breakfast omission adversely influenced the clock gene expression and significantly increased glycemic response after lunch. It suggests a high relevance of breakfast consumption on the clock gene regulation of postprandial glycemia [43].

**Figure 4:**
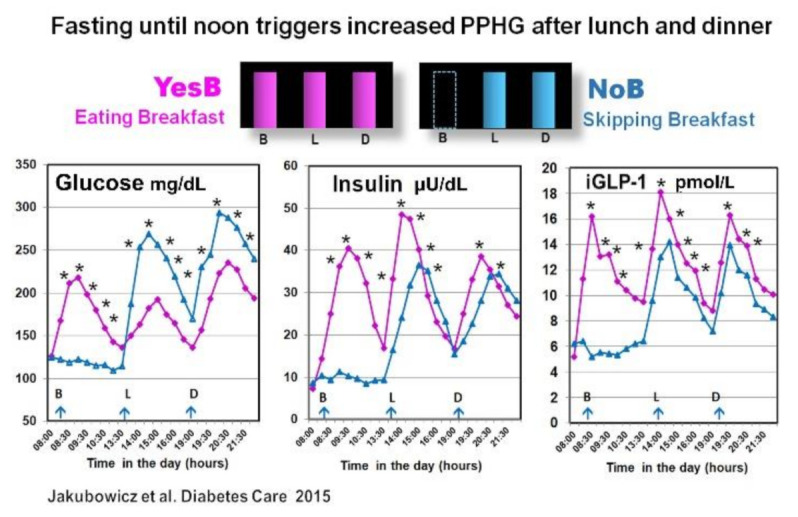
All day graphs for glucose, insulin, and intact GLP-1 (iGLP-1) postprandial responses, after breakfast, lunch and dinner. Statistics were between the NoB and the YesB of the same meal. * *p* < 0.001. B: Breakfast; L: Lunch; D: Dinner. “Reproduced and adapted with permission” [56].

In another crossover study in T2D patients, we reported that the omission of breakfast (NoB) versus breakfast consumption (YesB), was associated with significantly higher glycemia response, after lunch and also after subsequent dinner. Moreover, compared to the day when breakfast was consumed, the omission of breakfast led to reduced and delayed insulin, C-peptide, and iGLP-1 responses after lunch and dinner [56] (Figure 4).

The reduction of postprandial glycemia and higher and faster insulin response after lunch with prior breakfast consumption in YesB day, was previously reported in healthy and in T2D individuals [35,43,75,78,79], and was described as the second meal phenomenon [35,75,78,79]. It has been reported that previous breakfast consumption may enhance β-cell memory and β-cell responsiveness at the second meal (lunch) [80].

However, in our study this effect of breakfast was extended to dinner [56]. Indeed, the absence of breakfast led to higher postprandial glucose and decreased GLP-1 and insulin response after lunch and also after dinner [56]. It has been suggested that fasting until noon on NoB day may reduce the β-cell memory and β-cell responsiveness in an extended fashion, resulting in less and delayed postprandial insulin response after both lunch and dinner [56,81].

The explanation is based on a recent report showing that nutrient depletion or starvation induces lysosomal degradation of nascent insulin secretory granules and to less β-cell secretory granule biogenesis [81]. It leads to deficient and delayed postprandial insulin response extended to lunch and dinner [56]. The increased postprandial GLP-1 on the YesB day is also associated with enhanced β-cell memory [82]. Further, it may explain the reduction of glycemic excursions after lunch and dinner on the day when breakfast was consumed [56] (Figure 4). 

Moreover, the breakfast consumption triggers correct oscillatory clock gene expression, namely *BMAL1*, RORα, PER1, and *AMPK* [43], and the clock gene regulation of glucose metabolism, thereby improving the postprandial GLP-1, insulin, and glucose responses after subsequent meals [43,56,79].

The upregulation of *AMPK*, in YesB day, significantly enhances GLUT-4 translocation, muscular glucose uptake, and postprandial insulin response further reducing post meal glycemic excursions [67]. *AMPK* is positively linked to *SIRT1*, and its beneficial effects on insulin sensitivity, β-cell proliferation and viability [67,68,69,70]. 

Breakfast consumption is essential when targeting glycemic control in T2D. The upregulation of the clock genes induced by breakfast consumption positively influences cardiovascular activity, heart rate, blood pressure, adipose tissue, and other metabolic organs [83]. Therefore, breakfast consumption may improve overall metabolism and reduce cardiometabolic complications of T2D.

### 3.2. High Energy Breakfast Diet “Breakfast Diet” (Bdiet) Reduces overall Postprandial Glycemia and Body Weight in Metabolic Syndrome

Studies in rodents and humans suggest that not only the amount but also the hour of food intake, especially the time of energy, protein, and CH intake, play an essential role in the circadian clock regulation of energy, and glucose homeostasis, thereby influencing the glycemic postprandial excursions [26,42,44,48,49,50,51]. Several reports showed that ingested calories are more efficiently used in the morning than at dusk [26], and this is evidenced by less hyperglycemic excursions throughout the day and better weight loss, when most of energy and CH are assigned to the early hours of the day, compared to iso-energetic calorie and CH intake mainly in the evening [35,39,84].

**Figure 5:**
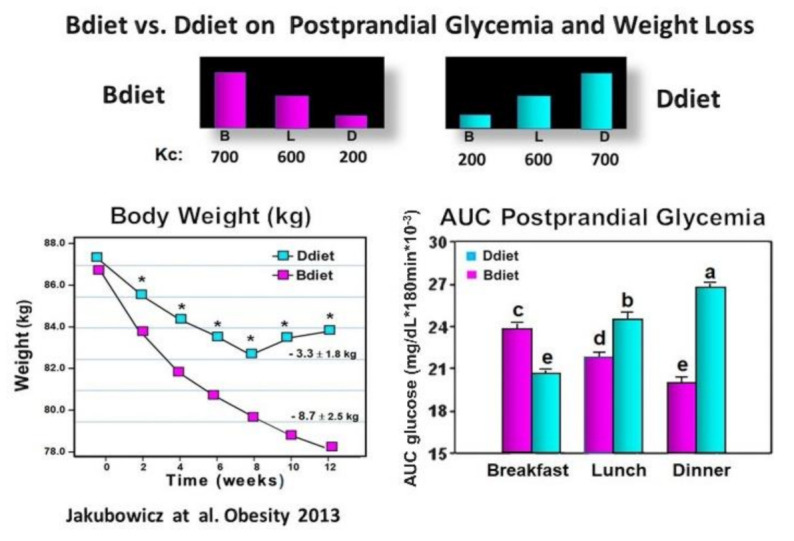
Effect of Bdiet versus Ddiet on postprandial glycemia and weight loss in metabolic syndrome. In the upper part is shown the caloric content for Breakfast (B), Lunch (L), and Dinner (D) in Bdiet and Ddiet. The line chart shows the changes in body weight, recorded every two weeks during the 12 weeks of the study in Bdiet and Diet groups. The bar graph illustrates AUC for postprandial glycemia calculated at 0–180 min after breakfast, lunch, and dinner meal challenges, of the assigned diet. The meal tests were performed on a single day during the second week of the study. Values are means ± SE; Bdiet–breakfast diet group; Ddiet—dinner diet group; Asterisks denotes *p* < 0.05; Bars with different letters, denote significant difference *p* < 0.05.“Reproduced and adapted with permission” [39].

We examined in participants with metabolic syndrome whether a diet with overall similar daily caloric intake but with different meal timing and distribution: either consuming a high-energy and CH breakfast (Bdiet) or high-energy and CH dinner (Ddiet) has a distinct influence on the glycemic postprandial response after breakfast, lunch, and dinner. We also explored the influence of Bdiet vs. Ddiet on overall glycemia, weight loss, and appetite scores. The energy distribution of Bdiet was: large breakfast (700 kcal, 50%), medium-sized lunch (600 kcal, 36%), and small dinner (200 kcal, 14%). In Ddiet, the plan was reversed; small breakfast and large dinner [39] (Figure 5).

Over 12 wk. of the study, the body weight decreased significantly in both groups. However, the Bdiet group showed a 2.5-fold more significant weight loss (Figure 5). After the high-calorie dinner meal test in the Ddiet group, the postprandial glucose response was significantly higher compared to the postprandial glucose response to the isocaloric high-calorie breakfast meal in the Bdiet group (Figure 5). The overall postprandial response to breakfast, lunch, and dinner challenge meals, expressed as overall AUC for postprandial glycemia, was significantly lower in the Bdiet group than the Ddiet group [39]. These results are in line with several recent reports suggesting metabolic disadvantages of high energy and CH consumption in evening hours, while high energy and CH meals consumed in the early hours of the day, may reduce the insulin resistance and glucose post meal response in obese and prediabetics [29,39,40,77].

A high-energy and CH breakfast (Bdiet) is more beneficial than a high-energy and CH dinner to reduce overall postprandial glycemia. Avoiding high energy and CH intake at dusk and in the evening may be advantageous, particularly for lowering postprandial glycemic excursions, and may reduce the risk of T2D and cardiovascular diseases.

### 3.3. High Energy Breakfast Diet “Breakfast Diet” (Bdiet) Versus High Energy Dinner Diet (Ddiet) Reduces overall PPHG in Type 2 Diabetes

**Figure 6:**
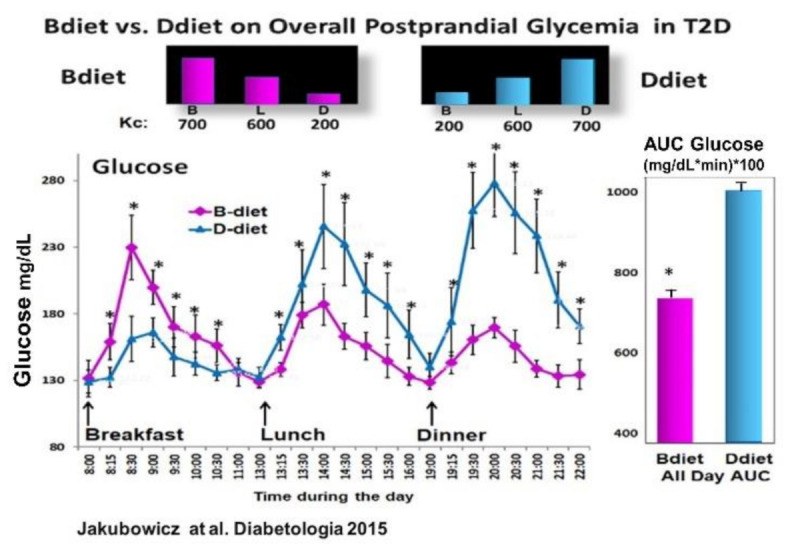
Effect of Bdiet vs. Ddiet on overall postprandial hyperglycemia (PPHG) in T2D. All-day graph for glucose and AUC for all day postprandial glycemia in Bdiet vs. Ddiet group. * *p* < 0.05 “Reproduced and adapted with permission” [35].

Based on the previous study [39], in obese non-diabetic individuals, showing that Bdiet schedule vs. Ddiet significantly reduced overall postprandial glycemia; and on the studies reporting that breakfast consumption vs. skipping breakfast in T2D lead to reduced PPHG and greater and faster postprandial insulin and GLP-1 responses and after subsequent meals [43,56]; we further tested in T2D patients whether the Bdiet schedule reduces overall postprandial glycemia by enhancing prandial GLP-1 and insulin responses compared to the Ddiet schedule [35] (Figure 6).

Compared to the Ddiet schedule, the Bdiet led to significantly reduced overall PPHG and glucose excursions (Figure 6). Further, the Bdiet schedule significantly increased the integrated AUC for the postprandial responses of insulin, C-peptide, intact GLP-1, and total GLP-1 along the entire day compared to the Ddiet schedule [35]. Although both diets Bdiet and Ddiet, were isocaloric and with the same composition, the difference in meal timing and distribution led to a significant reduction in overall PPHG and glucose excursions in the Bdiet compared to Ddiet.

### 3.4. High Energy Breakfast Diet “Breakfast Diet” (3Mdiet-Bdiet) Versus Traditional Six Meals Diet (6Mdiet) Reduces overall Glycemia, Body Weight and Insulin Dose Requirements in Type 2 Diabetes

More recently, in insulin treated T2D, we explored the effects of either one of two isocaloric diet interventions (DI) with different meal timing and distribution during three months. One DI was aligned to the circadian clock, similar to the Bdiet of our previous studies described above, with three meals a day consisted of high energy and CH breakfast and low in energy and CH dinner (3Mdiet-Bdiet). The other DI was the traditional diet consisting of six small meals with energy and CH evenly distributed along the day, without any temporal alignment to the rhythms imposed by the circadian clock (6Mdiet).

Compared to the traditional diet (6Mdiet), the 3Mdiet-Bdiet schedule led to a significant resetting effect in the oscillatory expression of clock genes linked to the regulation of muscular glucose uptake, hepatic glucose production and insulin secretion, namely *BMAL1*, CRY1, PER2, RORα. Likewise, 3Mdiet-Bdiet led to an increase of daily *SIRT1* levels [15]. This enhanced clock gene synchronization in the 3Mdiet was associated with a greater reduction of HbA1c, weight loss, fasting glucose, and glycemic excursion evaluated using continuous glucose monitoring (CGM).

The design of this study doesn’t allow the direct assessment of postprandial glycemic response after each meal. However, the significantly higher glycemic excursions in 6Mdiet vs. 3Mdiet-Bdiet measured by CGM during hours assigned to meals (i.e., CGM segment from 06:00 to 22:00) are highly suggestive of increased overall postprandial glycemic responses in the traditional 6Mdiet compared to 3Mdiet-Bdiet.

**Figure 7:**
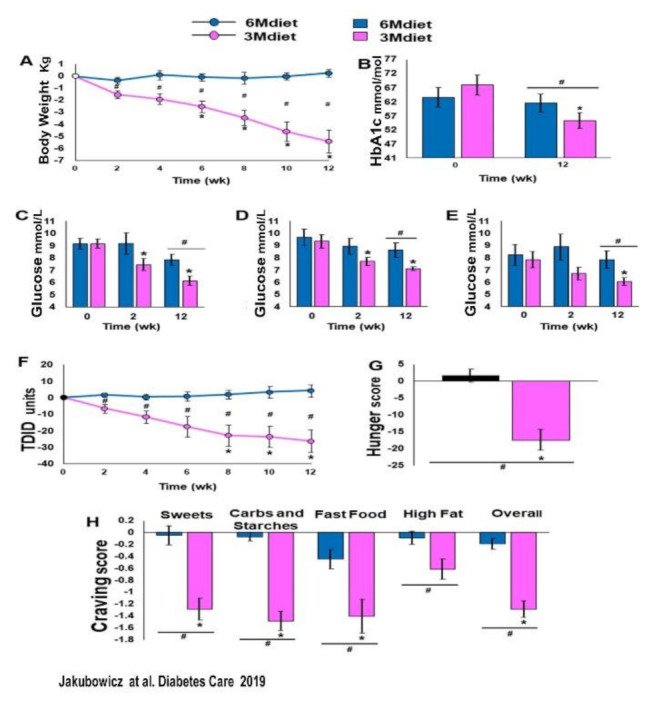
Body weight, HbA1c, glucose levels, TDID, hunger, and cravings at baseline, 2 weeks and 12 weeks of 3Mdiet and 6Mdiet. (**A**): Weight loss. (**B**): HbA1c. (**C**): Fasting glucose. (**D**): Twenty-four–hour mean glucose. (**E**): Nocturnal (0000–0600 h) mean glucose. (**F**): Total Daily Insulin Dose (TDID). (**G**): Hunger scores. (**H**): Mean daily craving scores. Values are mean ± SE. * Significant difference within groups compared with baseline, *p* < 0.05; # significant difference between groups, *p* < 0.05. ■:3Mdiet-Bdiet ■:6Mdiet “Reproduced and adapted with permission” [15].

**Figure 8:**
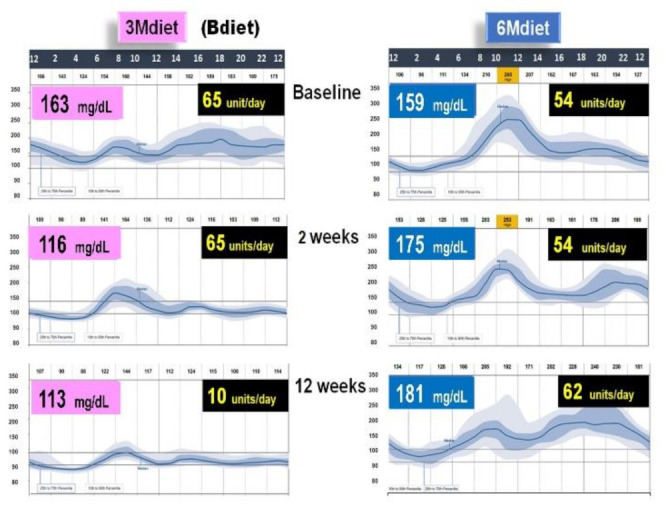
Graphical illustration of Continuous Glucose Monitoring (CGM) of representative patient from a group assigned to 3Mdiet-Bdiet and to 6Mdiet. On the right side of both graphs are the values of overall mean glucose (mg/dL), assessed by CGM, at baseline, after two weeks and 12 weeks of the diet intervention. On the right side of both graphs is shown the insulin dose (units/day) required at baseline after two weeks and 12 weeks of the diet intervention. This graph is based on the results from [15].

These results are consistent with previous reports showing that misaligned meal patterns like skipping breakfast or the traditional diet consisting on frequent small meals, including snacking at night, promote asynchrony of clock gene expression and metabolic disturbances, i.e., weight gain and hyperglycemia. [13,42,43,44,45,46,47,48,49,50,51]. In contrast, greater intake in the morning than in the evening has a resetting effect on clock gene oscillation [15,38,43]. It is associated with beneficial effects on weight loss, glycemia, and appetite control, independent of total energy intake [30,39,40,41,42,43,44,45,46,47,73,74,75,76,77,84] (Figure 7 and Figure 8).

Notably, the reduced overall glycemic excursions in 3Mdiet-Bdiet, were also significantly reduced during the nocturnal segment, suggesting an improvement in the nocturnal hepatic insulin sensitivity and reduced glucose production in 3Mdiet-Bdiet [24]. The time spent in the normal glucose range was also significantly increased in 3Mdiet-Bdiet than 6Mdiet, while the percentage of time spent in hyperglycemia was significantly reduced [15].

Importantly the titration of total daily insulin dose 3Mdiet-Bdiet group resulted in a significant reduction in insulin requirement by 27.5 units (Figure 8). Also, we found that the appetite and craving scores for all kinds of foods, but especially for sweets, were all significantly reduced only in the 3Mdiet-Bdiet.

We can assume in this study [15] that meal timing aligned to the circadian clock by shifting most calories and CH to the early hours of the day upregulated the pivotal clock gene oscillatory mRNA expression. The upregulation of clock gene expression might be the essential mechanism of the beneficial effect on weight loss, glycemic control, and appetite, achieved with a diet intervention aligned to the circadian clock [15].

### 3.5. Addition of Whey Protein to High Energy Breakfast (Bdiet), Enhance the Reduction Postprandial Hyperglycemia and Body Weight in Type 2 Diabetes

As we described above, the Bdiet schedule resulted in a significant reduction in overall postprandial glycemia and weight reduction in obese non-diabetics [35,40] and T2D individuals [15,40,56]. Bdiet also led in T2D individuals to a significant decrease in HbA1c [74]. The lowering effect of the Bdiet on the overall PPHG was associated with a greater and earlier increase in GLP-1 and insulin responses after breakfast, lunch, and dinner, suggesting a day-long effect of Bdiet [35,56,75].

It has been reported that increased protein (>35 g) intake in the breakfast leads to the reduction of all-day postprandial glycemic excursions [85,86] and greater GLP-1 and insulin and response after breakfast, lunch, and dinner [35,56].

In addition to the protein load, the source and quality of the protein ingested in the breakfast are critical for its lowering effect on the glycemic postprandial response [87,88]. It was showed in previous studies that Whey protein exerts a greater lowering effect on postprandial glucose compared to other proteins such as eggs, soy, gluten, fish, or casein in healthy [89,90,91] and T2D individuals [76,92,93,94].

Particularly, Whey milk protein that accounts for 20% of whole milk protein has insulinotropic/β-cell-stimulating and glucose-lowering effects through bioactive peptides and amino acids generated during its gastrointestinal digestion [89,90].

These bioactive peptides can stimulate several gut hormones, including GLP-1, which stimulate β-cells insulin secretion [89,92,93,94,95], and reduce the activity of DPP-4, attenuating the degradation of GLP-1 in the proximal gut [94]. Hence, Whey protein pre-load breakfast may be a potential mode to stimulate GLP-1 secretion, thereby augmenting the early insulin response and reducing postprandial glucose.

**Figure 9:**
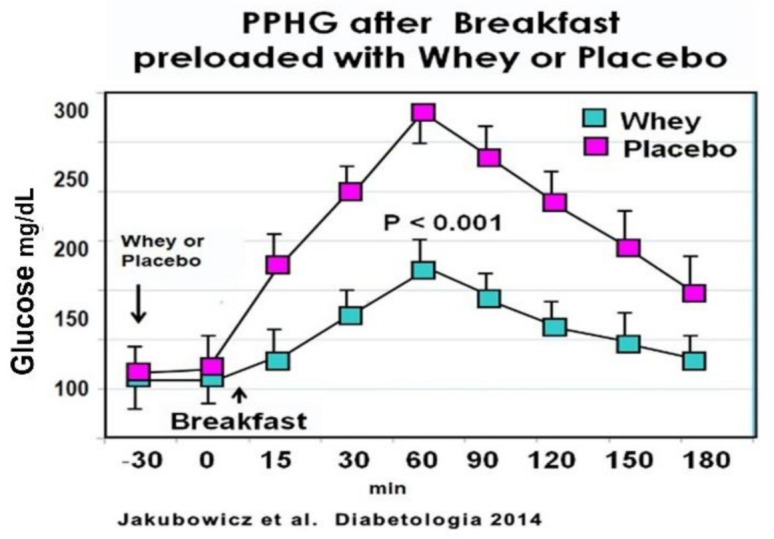
Meal tolerance test with Whey or placebo preload. Participants consumed a pre-meal 250 mL drink of either 50 g Whey protein concentrate or water (placebo). Blood samples were taken immediately before the preload (t = −30 min) and every 30 min after that. Participants were served a high-glycaemic index breakfast instantly after the second blood sample (t = 0). For every time point, blood samples were analyzed for glucose. Values are means ± SEM, n = 15, * *p* < 0.05 vs. placebo for the same time point. “Reproduced and adapted with permission” [76].

In an acute crossover study, T2D patients consumed a high glycemic index breakfast, one day preloaded with a drink containing 50 g of Whey protein and other day preloaded with water. Breakfast preloaded with Whey protein vs. water displayed a significant reduction in postprandial glucose response [76] (Figure 9). Furthermore, the addition of Whey pre-load before breakfast led to a substantial increase of postprandial GLP-1 response, predominantly during the early interval, and significantly stimulated the early insulin response and the insulin post-breakfast peak [76] (Figure 9).

These results are in line with previous reports showing that Whey protein pre-load exerts a potent stimulatory effect on β-cell secretion, reducing postprandial glycemia in healthy [79,80,93,94] and T2D patients [92,94]. This enhanced and almost restored early insulin secretion after Whey pre-load is important since a deficiency or loss of this early insulin response is a key abnormality contributing to hyperglycemia and T2D [4,5,6].

The increase of the postprandial GLP-1 after Whey pre-load occurred in a parallel fashion with the insulinotropic effect. This correlation supports that the higher incretin response is the mechanism subserving the more rapid and higher insulin response in the Whey protein group.

Whey protein pre-load of high glycemic index breakfast stimulated the postprandial total and intact GLP-1 responses, the early prandial insulin secretion, and significantly reduced the PPHG in T2D patients. Therefore, Whey protein may represent a novel glucose-lowering strategy in T2D [76].

**Figure 10:**
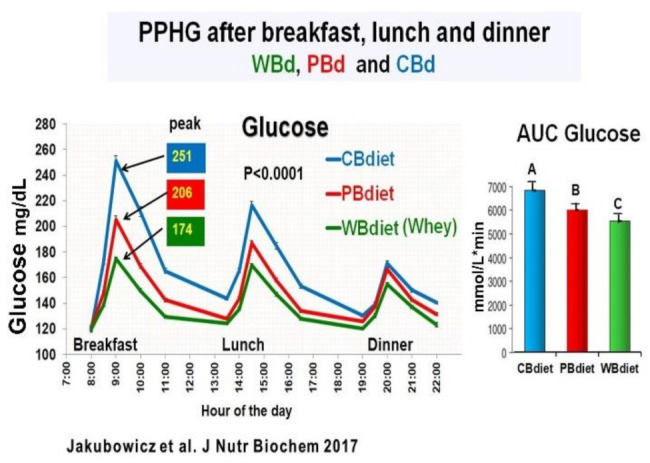
All day line chart for PPHG after breakfast, lunch and dinner; and daily AUC for Postprandial Glucose. The three diet intervention groups were: Whey protein breakfast (WBdiet) group; protein breakfast (**PBdiet**) group and carbohydrate breakfast (**CBdiet**) group. Different letters denote significant difference, *p* < 0.05. “Reproduced and adapted with permission” [75].

We further explored in T2D patients the long-term effect of Whey protein. In this study in T2D, we tested during 12 weeks the long-term influence of Whey protein on weight loss, reduction of overall PPHG, and HbA1c. We used a well-established feeding regimen (Bdiet) consisting of a high-calorie and protein breakfast, medium-sized lunch, and low-calorie dinner [35,39,56,74] (Figure 10).

The participants were randomly assigned to one of the three diet intervention groups The only difference among the three diet interventions was the breakfast composition: (1) Whey protein breakfast diet (WBdiet), with high protein content at breakfast, containing Whey as the primary source of protein; (2) Protein breakfast diet (PBdiet), with high protein content at breakfast from other sources, i.e., eggs, tuna, soy; and (3) Carbohydrate breakfast diet (CBdiet), with low protein and high CH content in the breakfast [75]. All patients underwent 3 all-day meal challenges testing WBdiet, PBdiet, and CBdiet (Figure 10).

Although the three diet interventions (CBdiet, PBdiet, WBdiet) had similar lunch and dinner composition, the effect during a meal challenge on postprandial glycemia, insulin, GLP-1, ghrelin, glucagon, and appetite scores was not limited to breakfast but extended to subsequent meals, i.e., lunch and dinner [75].

Compared to PBdiet and CBdiet, the Whey in the breakfast group (WBdiet) showed the lowest overall PPHG and overall AUC for postprandial plasma glucose, ghrelin, and hunger, and highest overall AUC for postprandial plasma insulin, C-peptide, intact GLP-1, and satiety scores. PBdiet showed similar benefits than WBdiet but less pronounced. WBdiet also led to a greater reduction of HbA1c and body weight compared to the other groups. The greatest reduction of PPHG was achieved after breakfast containing Whey (WBdiet) is supported by the increased GLP-1 and its insulinotropic effect, as reported in healthy and T2D individuals [25,80,81,89,94] (Figure 10).

Whey protein and its main components, i.e., alpha-lactalbumin, β-lactoglobulin, and the bioactive peptides generated during its gastrointestinal digestion, are potent secretagogues of L-cells, increasing GLP-1 intracellular levels and production [25,94,95,96]. Whey protein also activates mTOR in the intestinal L cells, increasing prandial GLP-1 secretion, further stimulating β-cell postprandial response [94,95,96]. The increased GLP-1 release in WBdiet, might explain the beneficial effect of Whey consumption on PPHG and enhanced insulin release at subsequent meals after breakfast [25,80,81,94]. Whey protein ingestion is also associated with a rapid increase in plasma amino acid concentrations, specifically leucine, isoleucine, and valine, exerting a potent direct insulinotropic/β-cell-stimulating effect [89,94].

This study showed that in T2D individuals, a diet consisting of high-energy breakfast, medium-sized lunch, and reduced energy dinner is more beneficial in reducing overall PPHG, body weight, and HbA1c levels, when the primary protein source at breakfast is Whey, indicating that Whey protein at breakfast might be a potent adjuvant for the management of type 2 diabetes [75].

## 4. Conclusions Remarks

Postprandial hyperglycemia in T2D leads to a progressive decline of β-cell function and increases the cardiovascular risk in T2D [2,6,7]. Therefore, DI meal timing and composition should focus on mitigating glycemic peaks to reduce the decline of β-cell function and prevent cardiovascular complications [4].

Almost all hormonal and enzymatic processes influencing the PPHG, like muscular glucose uptake, the secretion of GLP-1, and β-cell insulin production, are regulated by the circadian clock and display circadian rhythms [17,18,19,20,21,22,23,24]. The circadian clock is synchronized to the day/night cycle and food cues, namely, meal timing and food availability [13,14].

As a consequence of circadian clock regulation, the β-cell secretion and responsiveness, insulin sensitivity, and muscular glucose uptake are enhanced in the early hours of the day compared to afternoon and night [8,16,17,18,19,20,21,22,23,25,26,27,28,29,30,31,32,33]. Therefore, the metabolism is optimal for food intake in the morning (i.e., breakfast). In contrast, the evening and nighttime are optimal for fasting and sleep [9,34,35,36,37].

Meal timing, independently of the total energy intake, exerts a critical influence on peripheral clocks genes involved in regulating metabolic processes and PPHG [41,42,43,44,45,46,47]. Several recent reports suggested metabolic disadvantages of reduced energy breakfast and high energy and CH consumption in evening hours. While high energy and CH consumption shifted into morning hours, “high energy breakfast” (Bdiet) increased the weight loss, insulin sensitivity and reduced the overall postprandial glycemia in obese and prediabetics [39,40,77], and substantially decrease the PPHG and HbA1c in diabetic individuals [15,35,74,75]. Moreover, in T2D, the omission of breakfast disrupts circadian clock gene expression and is linked to worsening of PPHG and delayed and deficient early insulin and GLP-1 response after subsequent meals [15,43,49].

In contrast, meal-timing pattern, aligned with the circadian clock, consuming high energy and CH breakfast (Bdiet) exerts a powerful synchronizing effect on pivotal clock gene expression. It leads to a significant reduction of postprandial glycemic peaks across the day and enhanced insulin, C-peptide, and GLP-1 postprandial responses in healthy and T2D patients. Therefore, breakfast consumption is critical for achieving metabolic homeostasis and improving PPHG in T2D [15,30,35,73,74].

Synchronization of the clock gene expression through a diet intervention consisting of meal timing aligned to the circadian clock by shifting more calories and CH to the early hours of the day (Bdiet) is a promising strategy for therapeutic interventions to improve PPHG, weight loss, and to prevent cardiometabolic complication in type 2 diabetes.

## Data Availability

Not applicable.

## References

[1] Monnier L., Lapinski H., Colette C. (2003). Contributions of fasting and postprandial plasma glucose increments to the overall diurnal hyperglycemia of type 2 diabetic patients: Variations with increasing levels of HbA1c. Diabetes Care.

[2] Ceriello A., Colagiuri S., Gerich J., Tuomilehto J. (2008). Guideline for management of postmeal glucose. Nutr. Metab. Cardiovasc. Dis..

[3] Szuszkiewicz-Garcia M.M., Davidson J.A. (2014). Cardiovascular disease in diabetes mellitus: Risk factors and medical therapy. Endocrinol. Metab. Clin. N. Am..

[4] Bruce D.G., Chisholm D.J., Storlien L.H., Kraegen E.W. (1988). Physiological importance of deficiency in early prandial insulin secretion in non-insulin-dependent diabetes. Diabetes.

[5] Kahn S.E. (2001). The Importance of β-Cell Failure in the Development and Progression of Type 2 Diabetes. J. Clin. Endocrinol. Metab..

[6] Del Prato S. (2003). Loss of early insulin secretion leads to postprandial hyperglycaemia. Diabetologia.

[7] Fonseca V. (2003). Clinical significance of targeting postprandial and fasting hyperglycemia in managing type 2 diabetes mellitus. Curr. Med. Res. Opin..

[8] Oike H. (2017). Modulation of circadian clocks by nutrients and food factors. Biosci. Biotechnol. Biochem..

[9] Poggiogalle E., Jamshed H., Peterson C.M. (2018). Circadian regulation of glucose, lipid, and energy metabolism in humans. Metabolism.

[10] Panda S. (2016). Circadian physiology of metabolism. Science.

[11] Oosterman J.E., Kalsbeek A., La Fleur S.E., Belsham D.D. (2015). Impact of nutrients on circadian rhythmicity. Am. J. Physiol. Regul. Integr. Comp. Physiol..

[12] Koronowski K.B., Sassone-Corsi P. (2021). Communicating clocks shape circadian homeostasis. Science.

[13] Javeed N., Matveyenko A.V. (2018). Circadian etiology of type 2 diabetes mellitus. Physiology.

[14] Froy O., Garaulet M. (2018). The circadian clock in white and brown adipose tissue: Mechanistic, endocrine, and clinical aspects. Endocr. Rev..

[15] Jakubowicz D., Landau Z., Tsameret S., Wainstein J., Raz I., Ahren B., Chapnik N., Barnea M., Ganz T., Menaged M. (2019). Reduction in Glycated Hemoglobin and Daily Insulin Dose Alongside Circadian Clock Upregulation in Patients with Type 2 Diabetes Consuming a Three-Meal Diet: A Randomized Clinical Trial. Diabetes Care.

[16] Jordan S.D., Lamia K.A. (2013). AMPK at the crossroads of circadian clocks and metabolism. Mol. Cell. Endocrinol..

[17] Yoshino J., Imai S.I. (2010). A clock ticks in pancreatic β cells. Cell Metab..

[18] Sadacca L.A., Lamia K.A., DeLemos A.S., Blum B., Weitz C.J. (2011). An intrinsic circadian clock of the pancreas is required for normal insulin release and glucose homeostasis in mice. Diabetologia.

[19] Rakshit K., Qian J., Ernst J., Matveyenko A.V. (2016). Circadian variation of the pancreatic islet transcriptome. Physiol. Genom..

[20] Gil-Lozano M., Mingomataj E.L., Wu W.K., Ridout S.A., Brubaker P.L. (2014). Circadian secretion of the intestinal hormone GLP-1 by the rodent L cell. Diabetes.

[21] Dyar K.A., Ciciliot S., Wright L.E., Biensø R.S., Tagliazucchi G.M., Patel V.R., Forcato M., Paz M.I.P., Gudiksen A., Solagna F. (2014). Muscle insulin sensitivity and glucose metabolism are controlled by the intrinsic muscle clock. Mol. Metab..

[22] Prasai M.J., Mughal R.S., Wheatcroft S.B., Kearney M.T., Grant P.J., Scott E.M. (2013). Diurnal variation in vascular and metabolic function in diet-induced obesity: Divergence of insulin resistance and loss of clock rhythm. Diabetes.

[23] Saad A., Man C.D., Nandy D.K., Levine J.A., Bharucha A.E., Rizza R.A., Basu R., Carter R.E., Cobelli C., Kudva Y.C. (2012). Diurnal pattern to insulin secretion and insulin action in healthy individuals. Diabetes.

[24] Basu A., Joshi N., Miles J., Carter R.E., Rizza R.A., Basu R. (2018). Paradigm shifts in nocturnal glucose control in type 2 diabetes. J. Clin. Endocrinol. Metab..

[25] Lindgren O., Mari A., Deacon C.F., Carr R.D., Winzell M.S., Vikman J., Ahrén B. (2009). Differential islet and incretin hormone responses in morning versus afternoon after standardized meal in healthy men. J. Clin. Endocrinol. Metab..

[26] Ruddick-Collins L.C., Johnston J.D., Morgan P.J., Johnstone A.M. (2018). The Big Breakfast Study: Chrono-nutrition influence on energy expenditure and bodyweight. Nutr. Bull..

[27] Jamshed H., Beyl R.A., Manna D.L.D., Yang E.S., Ravussin E., Peterson C.M. (2019). Early time-restricted feeding improves 24-hour glucose levels and affects markers of the circadian clock, aging, and autophagy in humans. Nutrients.

[28] Bo S., Fadda M., Castiglione A., Ciccone G., De Francesco A., Fedele D., Guggino A., Parasiliti Caprino M., Ferrara S., Vezio Boggio M. (2015). Is the timing of caloric intake associated with variation in diet-induced thermogenesis and in the metabolic pattern? A randomized cross-over study. Int. J. Obes..

[29] Morgan L.M., Shi J.W., Hampton S.M., Frost G. (2012). Effect of meal timing and glycaemic index on glucose control and insulin secretion in healthy volunteers. Br. J. Nutr..

[30] Ravussin E., Beyl R.A., Poggiogalle E., Hsia D.S., Peterson C.M. (2019). Early Time-Restricted Feeding Reduces Appetite and Increases Fat Oxidation but Does Not Affect Energy Expenditure in Humans. Obesity.

[31] Gibbs M., Harrington D., Starkey S., Williams P., Hampton S. (2014). Diurnal postprandial responses to low and high glycaemic index mixed meals. Clin. Nutr..

[32] Nitta A., Imai S., Kajiyama S., Miyawaki T., Matsumoto S., Ozasa N., Hashimoto Y., Tanaka M., Fukui M. (2019). Impact of different timing of consuming sweet snack on postprandial glucose excursions in healthy women. Diabetes Metab..

[33] Van Cauter E., Shapiro E.T., Tillil H., Polonsky K.S. (1992). Circadian modulation of glucose and insulin responses to meals: Relationship to cortisol rhythm. Am. J. Physiol. Endocrinol. Metab..

[34] Scheer F.A.J.L., Hilton M.F., Mantzoros C.S., Shea S.A. (2009). Adverse metabolic and cardiovascular consequences of circadian misalignment. Proc. Natl. Acad. Sci. USA.

[35] Jakubowicz D., Wainstein J., Ahrén B., Bar-Dayan Y., Landau Z., Rabinovitz H.R., Froy O. (2015). High-energy breakfast with low-energy dinner decreases overall daily hyperglycaemia in type 2 diabetic patients: A randomised clinical trial. Diabetologia.

[36] Stenvers D.J., Jongejan A., Atiqi S., Vreijling J.P., Limonard E.J., Endert E., Baas F., Moerland P.D., Fliers E., Kalsbeek A. (2019). Diurnal rhythms in the white adipose tissue transcriptome are disturbed in obese individuals with type 2 diabetes compared with lean control individuals. Diabetologia.

[37] Morris C.J., Yang J.N., Garcia J.I., Myers S., Bozzi I., Wang W., Buxton O.M., Shea S.A., Scheer F.A.J.L. (2015). Endogenous circadian system and circadian misalignment impact glucose tolerance via separate mechanisms in humans. Proc. Natl. Acad. Sci. USA.

[38] Wehrens S.M.T., Christou S., Isherwood C., Middleton B., Gibbs M.A., Archer S.N., Skene D.J., Johnston J.D. (2017). Meal Timing Regulates the Human Circadian System. Curr. Biol..

[39] Jakubowicz D., Barnea M., Wainstein J., Froy O. (2013). High caloric intake at breakfast vs. dinner differentially influences weight loss of overweight and obese women. Obesity.

[40] Jakubowicz D., Froy O., Wainstein J., Boaz M. (2012). Meal timing and composition influence ghrelin levels, appetite scores and weight loss maintenance in overweight and obese adults. Steroids.

[41] Sherman H., Frumin I., Gutman R., Chapnik N., Lorentz A., Meylan J., le Coutre J., Froy O. (2011). Long-term restricted feeding alters circadian expression and reduces the level of inflammatory and disease markers. J. Cell. Mol. Med..

[42] Hatori M., Vollmers C., Zarrinpar A., DiTacchio L., Bushong E.A., Gill S., Leblanc M., Chaix A., Joens M., Fitzpatrick J.A.J. (2012). Time-restricted feeding without reducing caloric intake prevents metabolic diseases in mice fed a high-fat diet. Cell Metab..

[43] Jakubowicz D., Wainstein J., Landau Z., Raz I., Ahren B., Chapnik N., Ganz T., Menaged M., Barnea M., Bar-Dayan Y. (2017). Influences of Breakfast on Clock Gene Expression and Postprandial Glycemia in Healthy Individuals and Individuals with Diabetes: A Randomized Clinical Trial. Diabetes Care.

[44] Chaix A., Zarrinpar A., Miu P., Panda S. (2014). Time-restricted feeding is a preventative and therapeutic intervention against diverse nutritional challenges. Cell Metab..

[45] Mekary R.A., Giovannucci E., Willett W.C., Van Dam R.M., Hu F.B. (2012). Eating patterns and type 2 diabetes risk in men: Breakfast omission, eating frequency, and snacking. Am. J. Clin. Nutr..

[46] Nimitphong H., Siwasaranond N., Saetung S., Thakkinstian A., Ongphiphadhanakul B., Reutrakul S. (2018). The relationship among breakfast time, morningness–eveningness preference and body mass index in Type 2 diabetes. Diabet. Med..

[47] Reutrakul S., Hood M.M., Crowley S.J., Morgan M.K., Teodori M., Knutson K.L. (2014). The relationship between breakfast skipping, chronotype, and glycemic control in type 2 diabetes. Chronobiol. Int..

[48] Arble D.M., Bass J., Laposky A.D., Vitaterna M.H., Turek F.W. (2009). Circadian timing of food intake contributes to weight gain. Obesity.

[49] Wu T., Sun L., Zhuge F., Guo X., Zhao Z., Tang R., Chen Q., Chen L., Kato H., Fu Z. (2011). Differential roles of breakfast and supper in rats of a daily three-meal schedule upon circadian regulation and physiology. Chronobiol. Int..

[50] Fuse Y., Hirao A., Kuroda H., Otsuka M., Tahara Y., Shibata S. (2012). Differential roles of breakfast only (one meal per day) and a bigger breakfast with a small dinner (two meals per day) in mice fed a high-fat diet with regard to induced obesity and lipid metabolism. J. Circadian Rhythms.

[51] Sherman H., Genzer Y., Cohen R., Chapnik N., Madar Z., Froy O. (2012). Timed high-fat diet resets circadian metabolism and prevents obesity. FASEB J..

[52] Reinke H., Asher G. (2019). Crosstalk between metabolism and circadian clocks. Nat. Rev. Mol. Cell Biol..

[53] St-Onge M.P., Ard J., Baskin M.L., Chiuve S.E., Johnson H.M., Kris-Etherton P., Varady K. (2017). Meal Timing and Frequency: Implications for Cardiovascular Disease Prevention: A Scientific Statement from the American Heart Association. Circulation.

[54] Ando H., Ushijima K., Yanagihara H., Hayashi Y., Takamura T., Kaneko S., Fujimura A. (2009). Clock gene expression in the liver and adipose tissues of non-obese type 2 diabetic Goto-Kakizaki rats. Clin. Exp. Hypertens..

[55] Vieira E., Burris T.P., Quesada I. (2014). Clock genes, pancreatic function, and diabetes. Trends Mol. Med..

[56] Jakubowicz D., Wainstein J., Ahren B., Landau Z., Bar-Dayan Y., Froy O. (2015). Fasting Until Noon Triggers Increased Postprandial Hyperglycemia and Impaired Insulin Response after Lunch and Dinner in Individuals with Type 2 Diabetes: A Randomized Clinical Trial. Diabetes Care.

[57] Shimizu H., Hanzawa F., Kim D., Sun S., Laurent T., Umeki M., Ikeda S., Mochizuki S., Oda H. (2018). Delayed first active-phase meal, a breakfastskipping model, led to increased body weight and shifted the circadian oscillation of the hepatic clock and lipid metabolism-related genes in rats fed a high-fat diet. PLoS ONE.

[58] Kim Y.H., Lazar M.A. (2021). Transcriptional control of circadian rhythms and metabolism: A matter of time and space. Endocr. Rev..

[59] Pilorz V., Astiz M., Heinen K.O., Rawashdeh O., Oster H. (2020). The Concept of Coupling in the Mammalian Circadian Clock Network. J. Mol. Biol..

[60] Kuang J., Hou X., Zhang J., Chen Y., Su Z. (2014). Identification of insulin as a novel retinoic acid receptor-related orphan receptor α target gene. FEBS Lett..

[61] Johnston J.D. (2014). Physiological responses to food intake throughout the day. Nutr. Res. Rev..

[62] Sun C., Zhang F., Ge X., Yan T., Chen X., Shi X., Zhai Q. (2007). SIRT1 Improves Insulin Sensitivity under Insulin-Resistant Conditions by Repressing PTP1B. Cell Metab..

[63] Biancolin A.D., Martchenko A., Mitova E., Gurges P., Michalchyshyn E., Chalmers J.A., Doria A., Mychaleckyj J.C., Adriaenssens A.E., Reimann F. (2020). The core clock gene, Bmal1, and its downstream target, the SNARE regulatory protein secretagogin, are necessary for circadian secretion of glucagon-like peptide-1. Mol. Metab..

[64] Taira A., Arita E., Matsumoto E., Oohira A., Iwase K., Hiwasa T., Yokote K., Shibata S., Takiguchi M. (2019). Systemic oscillator-driven and nutrient-responsive hormonal regulation of daily expression rhythms for gluconeogenic enzyme genes in the mouse liver. Chronobiol. Int..

[65] Pérez-Mendoza M., Rivera-Zavala J.B., Díaz-Muñoz M. (2014). Daytime restricted feeding modifies the daily variations of liver gluconeogenesis: Adaptations in biochemical and endocrine regulators. Chronobiol. Int..

[66] Zhang E.E., Liu Y., Dentin R., Pongsawakul P.Y., Liu A.C., Hirota T., Nusinow D.A., Sun X., Landais S., Kodama Y. (2010). Cryptochrome mediates circadian regulation of cAMP signaling and hepatic gluconeogenesis. Nat. Med..

[67] Hardie D.G., Ross F.A., Hawley S.A. (2012). AMPK: A nutrient and energy sensor that maintains energy homeostasis. Nat. Rev. Mol. Cell Biol..

[68] Asher G., Gatfield D., Stratmann M., Reinke H., Dibner C., Kreppel F., Mostoslavsky R., Alt F.W., Schibler U. (2008). SIRT1 Regulates Circadian Clock Gene Expression through PER2 Deacetylation. Cell.

[69] Pinho A.V., Bensellam M., Wauters E., Rees M., Giry-Laterriere M., Mawson A., Ly L.Q., Biankin A.V., Wu J., Laybutt D.R. (2015). Pancreas-specific Sirt1-deficiency in mice compromises β-cell function without development of hyperglycemia. PLoS ONE.

[70] Nakahata Y., Sahar S., Astarita G., Kaluzova M., Sassone-Corsi P. (2009). Circadian Control of the NAD+ Salvage Pathway by CLOCK-SIRT1. Science.

[71] Wefers J., Van Moorsel D., Hansen J., Connell N.J., Havekes B., Hoeks J., Van Marken Lichtenbelt W.D., Duez H., Phielix E., Kalsbeek A. (2018). Circadian misalignment induces fatty acid metabolism gene profiles and compromises insulin sensitivity in human skeletal muscle. Proc. Natl. Acad. Sci. USA.

[72] Vetter C., Devore E.E., Ramin C.A., Speizer F.E., Willett W.C., Schernhammer E.S. (2015). Mismatch of sleep and work timing and risk of type 2 diabetes. Diabetes Care.

[73] Kahleova H., Belinova L., Malinska H., Oliyarnyk O., Trnovska J., Skop V., Kazdova L., Dezortova M., Hajek M., Tura A. (2014). Eating two larger meals a day (breakfast and lunch) is more effective than six smaller meals in a reduced-energy regimen for patients with type 2 diabetes: A randomised crossover study. Diabetologia.

[74] Rabinovitz H.R., Boaz M., Ganz T., Jakubowicz D., Matas Z., Madar Z., Wainstein J. (2014). Big breakfast rich in protein and fat improves glycemic control in type 2 diabetics. Obesity.

[75] Jakubowicz D., Wainstein J., Landau Z., Ahren B., Barnea M., Bar-Dayan Y., Froy O. (2017). High-energy breakfast based on whey protein reduces body weight, postprandial glycemia and HbA 1C in Type 2 diabetes. J. Nutr. Biochem..

[76] Jakubowicz D., Froy O., Ahrén B., Boaz M., Landau Z., Bar-Dayan Y., Ganz T., Barnea M., Wainstein J. (2014). Incretin, insulinotropic and glucose-lowering effects of whey protein pre-load in type 2 diabetes: A randomised clinical trial. Diabetologia.

[77] Sutton E.F., Beyl R., Early K.S., Cefalu W.T., Ravussin E., Peterson C.M. (2018). Early Time-Restricted Feeding Improves Insulin Sensitivity, Blood Pressure, and Oxidative Stress Even without Weight Loss in Men with Prediabetes. Cell Metab..

[78] Lee S.H., Tura A., Mari A., Ko S.H., Kwon H.S., Song K.H., Yoon K.H., Lee K.W., Ahn Y.B. (2011). Potentiation of the early-phase insulin response by a prior meal contributes to the second-meal phenomenon in type 2 diabetes. Am. J. Physiol. Endocrinol. Metab..

[79] Jovanovic A., Gerrard J., Taylor R. (2009). The second-meal phenomenon in type 2 diabetes. Diabetes Care.

[80] Korsgaard T.V., Colding-Jørgensen M. (2006). Time-dependent mechanisms in β-cells glucose sensing. J. Biol. Phys..

[81] Goginashvili A., Zhang Z., Erbs E., Spiegelhalter C., Kessler P., Mihlan M., Pasquier A., Krupina K., Schieber N., Cinque L. (2015). Insulin granules. Insulin secretory granules control autophagy in pancreatic β cells. Science.

[82] Efendic S., Portwood N. (2004). Overview of incretin hormones. Horm. Metab. Res..

[83] Richards J., Diaz A.N., Gumz M.L. (2014). Clock genes in hypertension: Novel insights from rodent models. Blood Press Monit..

[84] Garaulet M., Gómez-Abellán P., Alburquerque-Béjar J.J., Lee Y.C., Ordovás J.M., Scheer F.A.J.L. (2013). Timing of food intake predicts weight loss effectiveness. Int. J. Obes..

[85] Rains T.M., Leidy H.J., Sanoshy K.D., Lawless A.L., Maki K.C. (2015). A randomized, controlled, crossover trial to assess the acute appetitive and metabolic effects of sausage and egg-based convenience breakfast meals in overweight premenopausal women. Nutr. J..

[86] Park Y.M., Heden T.D., Liu Y., Nyhoff L.M., Thyfault J.P., Leidy H.J., Kanaley J.A. (2015). A high-protein breakfast induces greater insulin and glucose-dependent insulinotropic peptide responses to a subsequent lunch meal in individuals with type 2 diabetes. J. Nutr..

[87] Bendtsen L.Q., Lorenzen J.K., Larsen T.M., van Baak M., Papadaki A., Martinez J.A., Handjieva-Darlenska T., Jebb S.A., Kunešová M., Pfeiffer A.F. (2014). Associations between dairy protein intake and body weight and risk markers of diabetes and CVD during weight maintenance. Br. J. Nutr..

[88] Pasiakos S.M. (2015). Metabolic advantages of higher protein diets and benefits of dairy foods on weight management, glycemic regulation, and bone. J. Food Sci..

[89] Gunnerud U.J., Heinzle C., Holst J.J., Östman E.M., Björck I.M.E. (2012). Effects of pre-meal drinks with protein and amino acids on glycemic and metabolic responses at a subsequent composite meal. PLoS ONE.

[90] Nilsson M., Holst J.J., Björck I.M. (2007). Metabolic effects of amino acid mixtures and whey protein in healthy subjects: Studies using glucose-equivalent drinks. Am. J. Clin. Nutr..

[91] Frid A.H., Nilsson M., Holst J.J., Björck I.M.E. (2005). Effect of whey on blood glucose and insulin responses to composite breakfast and lunch meals in type 2 diabetic subjects. Am. J. Clin. Nutr..

[92] Ma J., Stevens J.E., Cukier K., Maddox A.F., Wishart J.M., Jones K.L., Clifton P.M., Horowitz M., Rayner C.K. (2009). Effects of a protein preload on gastric emptying, glycemia, and gut hormones after a carbohydrate meal in diet-controlled type 2 diabetes. Diabetes Care.

[93] Mignone L.E. (2015). Whey protein: The “whey” forward for treatment of type 2 diabetes?. World J. Diabetes.

[94] Jakubowicz D., Froy O. (2013). Biochemical and metabolic mechanisms by which dietary whey protein may combat obesity and Type 2 diabetes. J. Nutr. Biochem..

[95] Gillespie A.L., Calderwood D., Hobson L., Green B.D. (2015). Whey proteins have beneficial effects on intestinal enteroendocrine cells stimulating cell growth and increasing the production and secretion of incretin hormones. Food Chem..

[96] Xu G., Hong X., Tang H., Jiang S., Liu F., Shen Z., Li Z., Zhang W. (2015). Ghrelin regulates GLP-1 production through mTOR signaling in L cells. Mol. Cell. Endocrinol..

